# Isolated anal tuberculosis presenting as an anal fistula in an immunocompetent child

**DOI:** 10.1002/jpr3.70104

**Published:** 2025-10-27

**Authors:** Mariam Lagrine, Rabiy Elqadiry, Houda Nassih, Aicha Bourrahouat, Imane Ait Sab

**Affiliations:** ^1^ Department of Pediatrics University Hospital Center Mohammed VI Marrakech Morocco; ^2^ Child Health and Development Research Unit Cadi Ayyad University Marrakech Morocco

**Keywords:** *Mycobacterium tuberculosis*, perianal tuberculosis, previously healthy child

## Abstract

Extra‐pulmonary tuberculosis accounts for less than 15% of all tuberculosis cases, while intestinal tuberculosis accounts for less than 1% of extra‐pulmonary forms of the disease. Abdominal organ involvement is more common, but extension to the ano‐perineal region is extremely rare. We report a case of a 13‐year‐old child with an anal fistula without any other signs suggestive of tuberculosis. Diagnosis was confirmed by histopathological examination of the excised fistula and a positive GeneXpert test on fistula material. The initial work‐up ruled out Crohn's disease and other localizations secondary to tuberculosis, so the patient was put on anti‐tubercular drugs. Six months after the start of treatment, the lesion had completely disappeared, and no recurrence occurred after 8 months of follow‐up. Tuberculosis should generally be considered in the differential diagnosis of anal and perianal fistula despite the rarity of this location. In most cases, treatment is primarily medical, with surgical intervention reserved for complications such as abscesses or persistent fistulas.

## 1 INTRODUCTION

Tuberculosis is a widespread disease, with more than a third of the world's population infected. In 2022, an estimated 10.6 million people worldwide were infected with tuberculosis (TB), including 5.8 million men, 3.5 million women and 1.3 million children.^1^ Gastrointestinal TB accounts for less than 1% of the disease, with anoperineal TB being extremely rare.^2^ We report a case of anal tuberculosis in a 13‐year‐old immunocompetent child who presented with an anal fistula and no other associated sites.

## 2 CASE REPORT

A 13‐year‐old boy of Moroccan origin, with an unremarkable medical history and no family history of chronic inflammatory bowel disease or tuberculosis contacts, presented to the paediatric emergency department with anal pain and the appearance of a red, painful pustule that appeared 10 days earlier, followed 4 days later by purulent discharge. There were no other associated signs, particularly no digestive symptoms, arthralgia or ophthalmological signs.

**Figure 1:**
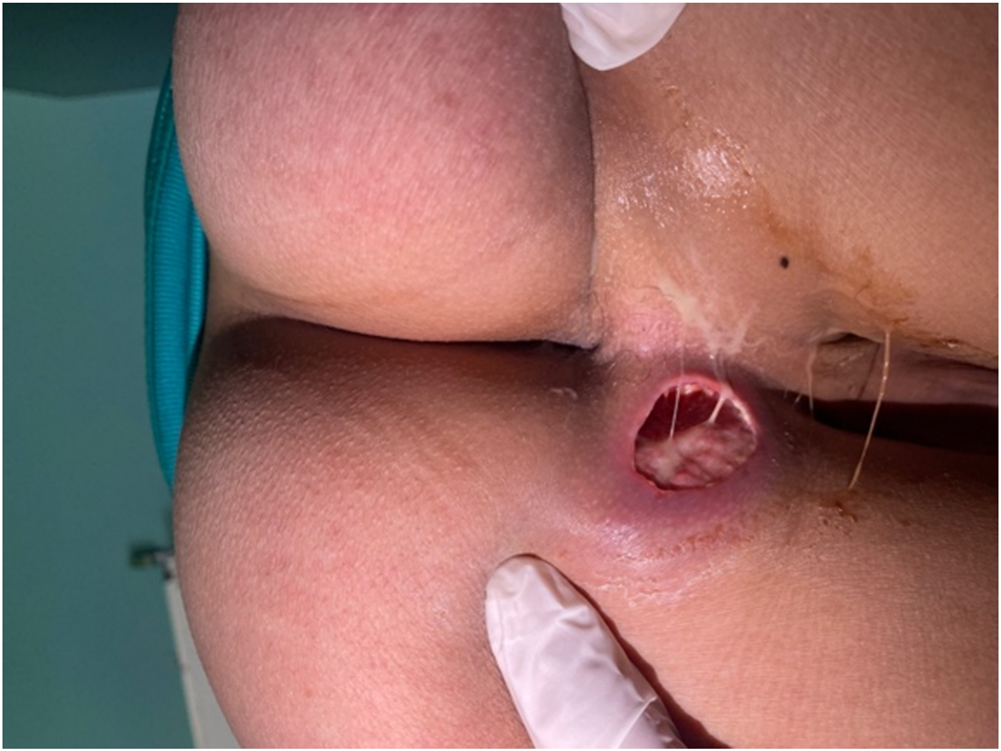
Anal fistula with purulent discharge.

On admission, clinical examination revealed a child with a stable haemodynamic and respiratory function: Heart rate (HR) = 73 bpm, respiratory rate (RR) = 18 cpm, temperature 36.7°C, weight: 42 kg (average for age), height: 156 cm (average for age). Anal examination revealed a 3‐cm fistula located to the left of the intergluteal fold with purulent discharge (Figure 1). The rest of the clinical examination was normal.

Biological tests showed 12,660 leukocytes with 72% neutrophils and a C‐reactive protein of 3.94 mg/L.

A plan was drawn up to exclude Crohn's disease and gastrointestinal tuberculosis. He underwent a colonoscopy, which was found to be normal. HIV serology and tuberculin skin test were negative. Interferon‐gamma release assay was not performed. His immunological tests, including c‐ANCA and p‐ANCA, were within normal limits. Pulmonary tuberculosis was ruled out by a normal chest X‐ray, performed in the absence of any respiratory symptoms or clinical findings suggestive of pulmonary involvement. No respiratory samples (sputum or gastric aspirates) were collected due to the absence of respiratory symptoms and the normal chest X‐ray. Abdominal ultrasound was performed and found to be normal with no lymphadenopathy or inflammatory thickening.

Pelvic MRI showed the presence of an active anoperineal fistula complicated by an abscess, classified as grade 4 on the St. James classification, with no intra‐digestive abscess collection, thickening, or adenopathy.

Local bacteriological sampling revealed the presence of an *Escherichia coli* strain resistant to ceftriaxone. GeneXpert was performed on the pus to search for *Mycobacterium tuberculosis* (BK). Ziehl‐Neelsen staining and culture were not performed due to limited sample volume. Histopathological examination of the excised fistula revealed an epithelioid granuloma with caseous necrosis.

**Figure 2:**
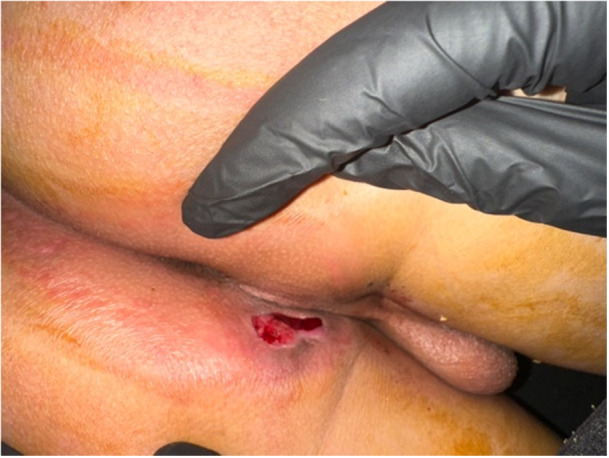
Progression after 3 months of anti‐tubercular treatment.

The child was initially treated with intravenous antibiotics (ciprofloxacin and amikacin), and then started on standard anti‐tubercular therapy consisting of a 2‐month intensive phase with isoniazid, rifampicin, pyrazinamide, and ethambutol, followed by a 4‐month continuation phase with isoniazid and rifampicin, totalling 6 months of treatment (Figure 2).

**Figure 3:**
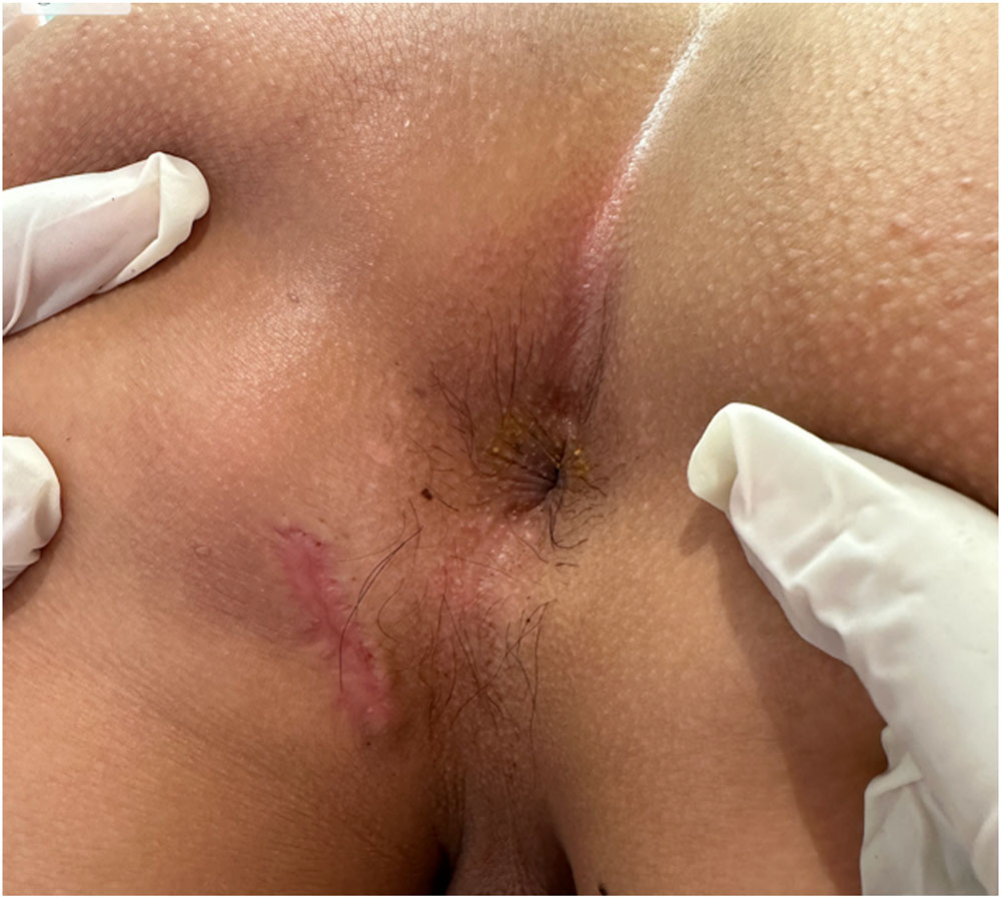
Complete cicatrization after anti‐tubercular treatment.

The outcome was very favourable, with no recurrence 8 months after completion of treatment (Figure 3).

## 3 DISCUSSION

Perianal tuberculosis is an exceptionally rare form of extrapulmonary TB, particularly in children, and there are few documented cases in the literature.^2^, ^3^, ^4^, ^5^ Historical surgical reports have also described cases of tuberculous fistula‐in‐ano, emphasizing the importance of considering tuberculosis in patients with fistulas that do not heal despite conventional treatment.^6^, ^7^

Tuberculosis remains a neglected cause of perianal sepsis or chronic infection. Sporadic cases of anal tuberculosis in adults have been described in the literature.^7^ Due to its nonspecific presentation, it often goes unrecognized and inadequately treated, leading to recurrence following routine surgical interventions.

The clinical presentation of anal tuberculosis may include pain, discharge, multiple or recurrent anal fistulas, and inguinal lymphadenopathy. These signs are not specific and can mimic other anorectal conditions. Diagnosis requires histopathological confirmation or detection of *M. tuberculosis* using nucleic acid amplification techniques such as GeneXpert, which has been shown to be significantly more effective than histology alone.^3^, ^8^ GeneXpert testing on pus samples demonstrates higher diagnostic accuracy than tissue samples.

A key step in confirming the tuberculous origin of anal fistulas is to rule out Crohn's disease and other gastrointestinal tuberculosis localizations, particularly since these conditions can share similar clinical and histopathological features. In our case, the absence of digestive symptoms, normal growth parameters, and unremarkable findings on colonoscopy, abdominal ultrasound, and pelvic MRI allowed us to confidently exclude both gastrointestinal TB and Crohn's disease.

In our patient, the absence of diarrhoea and growth delay, along with normal findings on abdominal ultrasound, colonoscopy, and magnetic resonance enterography, allowed us to exclude both gastrointestinal tuberculosis and Crohn's disease.

In some cases, anal tuberculosis may be associated with pulmonary or other extrapulmonary forms. However, several studies have documented primary anal fistulas without evidence of systemic involvement, supporting the possibility of isolated perianal tuberculosis. Therefore, TB should be considered in cases of recurrent or multiple anal fistulas unresponsive to conventional surgical treatment.

This is particularly relevant in paediatric cases, where isolated perianal TB may mimic more common anorectal disorders, leading to diagnostic delays.^4^, ^5^ A recent paediatric case described by Chaudhry et al. reported anal TB presenting as a fistula, underlining the importance of microbiological confirmation.^9^ In the largest study to date, GeneXpert testing on pus samples from anal fistulas demonstrated greater diagnostic accuracy than histopathology or culture alone.^8^

The management of tuberculous anal fistulas usually involves a combination of surgical drainage or fistulectomy to address suppuration and anti‐tubercular therapy to target the underlying infection. Most cases respond well to medical treatment alone and further surgery is rarely necessary if treatment is started early. In a study by Sultan et al., patients achieved favourable outcomes with anti‐TB therapy alone, without the need for repeated surgical intervention.^5^ These findings emphasize the importance of prompt diagnosis in preventing unnecessary procedures and ensuring complete recovery.

For paediatric patients, where a histological diagnosis may be difficult due to small biopsy samples and a low bacterial load, molecular techniques such as GeneXpert offer a sensitive and rapid alternative. This is particularly valuable for enabling early diagnosis and avoiding unnecessary delays in treatment.

This case reinforces the importance of including perianal tuberculosis in the differential diagnosis of chronic or recurrent anal fistulas, especially in endemic regions. Prompt diagnosis using both histopathological and molecular tools allows for early treatment and excellent outcomes without extensive surgical intervention.

## 4 CONCLUSION

Tuberculosis should be considered in cases of recurrent anal fistulae that do not respond to standard surgical treatment.^2^, ^5^ In our case, anti‐tubercular therapy led to complete healing within 6 months.

Given that clinical presentation is often nonspecific, histopathological examination of excised fistulous tissue, along with molecular tests such as GeneXpert, is essential for accurate diagnosis.^8^

Once tuberculosis is confirmed, early initiation of anti‐tubercular therapy is critical to achieving favourable outcomes and reducing the risk of recurrence.^5^

This case highlights the importance of including perianal tuberculosis in the differential diagnosis of persistent or atypical anal fistulas, especially in endemic regions or among high‐risk populations.^1^

## CONFLICT OF INTEREST STATEMENT

The authors declare no conflicts of interest.

## ETHICS STATEMENT

Written informed consent was obtained from the patient's legal guardian for publication of this case report and any accompanying images.
